# The Role of Ibuprofen and Midazolam in Pediatric Dentistry: A Retrospective Study and Neurophysiological Considerations

**DOI:** 10.3390/brainsci14111073

**Published:** 2024-10-28

**Authors:** Jan Rienhoff, Christian H. Splieth, Jacobus S. J. Veerkamp, Janneke B. Krikken, Sabine Rienhoff, Ulrike Halsband, Thomas Gerhard Wolf

**Affiliations:** 1 Pediatric Dental Practice, D-30177 Hannover, Germany; 2 Department of Preventive and Pediatric Dentistry, Center for Oral Health, Ernst Moritz Arndt University Greifswald, D-17475 Greifswald, Germany; 3 Kindertand, Pediatric Dental Practice, NL-1076 Amsterdam, The Netherlands; 4 Snoet Kindermondzorgcentrum, Pediatric Dental Practice, NL-1061 Amsterdam, The Netherlands; 5 Department of Psychology, Neuropsychology, University of Freiburg, D-79085 Freiburg, Germany; 6 Department of Restorative, Preventive and Pediatric Dentistry, School of Dental Medicine, University of Bern, CH-3010 Bern, Switzerland; 7 Department of Periodontology and Operative Dentistry, University Medical Center of the Johannes Gutenberg-University Mainz, D-55131 Mainz, Germany

**Keywords:** anxiety management, behavior management, clinical hypnosis, ibuprofen, midazolam, pediatric dentistry, sedation, post-treatment pain

## Abstract

**Background:** Managing anxiety and behavior during pediatric dental procedures is challenging. This study examines the effects of combining ibuprofen with midazolam sedation using both behavioral management and clinical hypnosis to improve patient cooperation and reduce post-treatment pain. **Methods:** A retrospective cohort study of 311 children (mean age 74.2 months, standard deviation (SD) = 24.7) was conducted. Patients received either midazolam with ibuprofen (n = 156) or midazolam only (n = 155). Behavior was assessed using the Venham Behavior Rating Scale and anxiety with the Dental Subscale of Children’s Fear Survey Schedule (CFSS-DS) and the Inventory of Stressful Situations (ISS) questionnaires. Statistical analyses included Mann–Whitney U tests and correlation analyses. **Results:** Ibuprofen did not significantly improve behavior during procedures (drinking phase 0.61, SD 1.31, *p =* 0.13; before treatment 0.25, SD 0.93, *p =* 0.53, anesthesia 1.21, SD 1.55, *p =* 0.29; after treatment 0.51, SD 1.22, *p =* 0.68), indicating that pharmacological pain management alone is insufficient to address behavioral challenges. Ibuprofen significantly reduced post-treatment pain, with 7.2% of cases reporting pain in the non-ibuprofen group compared to none in the ibuprofen group (*p <* 0.05). **Conclusions:** Ibuprofen had no effect on intraoperative behavior and only a limited effect on post-procedural pain, mainly for more invasive procedures. This study highlights the integration of sedation with behavioral strategies, such as clinical hypnosis, to manage anxiety and improve patient cooperation, aiming to enhance treatment outcomes using this integrative approach to pediatric dentistry. Further research is needed to optimize these strategies and verify them in a prospective setting.

## 1. Introduction

Treating children is generally considered a challenge that requires a lot of patience and creativity. Educational background and sex have a significant influence on dentists’ preferences when treating pediatric patients, with male dentists often feeling less comfortable treating children compared to their female colleagues or those with different educational experiences [1]. The main cause of disruptive behavior in young patients is dental fear, which is often related to early painful experiences and predicts uncooperative behavior [2,3]. The literature on children’s dental fear highlights the importance of understanding the fear acquisition process as set out in Rachman’s conditioning theory, which remains influential in this area [4,5]. Behavioral management techniques, which are critical to reducing anxiety, are effective when used appropriately, although they may be less effective with very young or cognitively impaired children who lack the necessary cognitive development [6,7]. With the declining acceptance of restraint techniques, such as the hand over the mouth and papoose board, among others in the Netherlands, Great Britain, and Scandinavian countries, the use of pharmacological support, including sedation and general anesthesia, has increased [8,9]. General anesthesia is often used to facilitate dental treatment in uncooperative children, although it is laborious and costly [10,11]. In some dental practices, pharmacological methods, such as general anesthesia, are used as a standard [12]. However, nitrous oxide and midazolam are commonly used sedatives in pediatric dentistry in a recommended combination of nitrous oxide (30–50%) and oral midazolam at a dosage of 0.75 mg/kg; however, the evidence for their use is weak [13]. Sedation can reduce a patient’s consciousness during medical procedures, while conscious sedation maintains the ability to keep the airway open and respond to stimuli [13]. Although nitrous oxide and dental hypnosis are also commonly used, their limitations, especially in very young or uncooperative patients, have led to the introduction of midazolam sedation [13,14,15,16]. The oral midazolam solution is recommended for sedation and hypnosis in children, as it is as effective as the injectable form and dexmedetomidine, and it is even more effective than ketamine at an optimal dose of oral midazolam of 0.5–1 mg/kg [17]. Midazolam offers advantages, such as rapid absorption and a shorter duration of action; although, its effect can be unpredictable. In the Netherlands, for example, low-dose oral sedation with midazolam is prescribed and used as light sedation, so previous safety rules, such as fasting or other preventive measures, can be omitted, and this method can be considered safe [18]. However, additional analgesics are needed to alleviate pain-related anxiety [19,20]. The effectiveness of combined hypnotic and pharmacological therapy in children undergoing dental treatment has not been well researched to date. This is remarkable since there is ample evidence of the side effects of medications used to alleviate pain and anxiety, which pose an increased risk for young children in particular. A study by Rienhoff et al. [16] showed that treatment with low-dose midazolam, combined with hypnosis techniques, is an effective option for children’s dental treatment. Previous studies and current research in children, adolescents, and adults [21,22,23,24] suggest that hypnosis is an effective method for reducing pain and anxiety during dental procedures. In a study by Huet et al. [23], children undergoing dental anesthesia were randomly assigned to either a hypnosis or a standard treatment group. Children in the hypnosis group were given targeted hypnotic suggestions based on stories and activities that matched the child’s interests during the anesthetic. The results showed that children under hypnosis experienced significantly less pain and anxiety than the control group. It is becoming increasingly clear that the best therapeutic outcomes are achieved when multiple therapeutic strategies are combined within a single session [25]. An integrative medical approach that includes clinical hypnosis, mindfulness, acupuncture, biofeedback, and yoga has the potential to significantly increase children’s well-being [25]. Recent studies are also investigating the effects of such interventions on the central nervous system [26]. It is particularly noteworthy that there is evidence that the use of hypnosis can reduce the need for medication by up to 40% [27]. Furthermore, the combination of ibuprofen and midazolam can have a synergistic effect in reducing pain and anxiety, with ibuprofen acting as an anti-inflammatory and analgesic and midazolam acting as a sedative and anxiolytic [28]. This combination is particularly useful in clinical contexts where pain and anxiety occur together, such as during surgery or dental treatment [28].

Given the limitations of existing sedation methods, this study aims to investigate whether combining midazolam with ibuprofen improves patient cooperation by reducing pain and anxiety during dental treatment. In this retrospective study, the child’s behavior was used as the primary outcome variable. This study also examines the correlations between anxiety levels, assessed using validated questionnaires (CFSS-DS and ISS) [29,30], and treatment outcomes [2]. Identifying these correlations could refine sedation strategies in pediatric dentistry, especially in populations with high dental anxiety and caries prevalence [31,32].

## 2. Materials and Methods

### 2.1. Study Design and Population

This retrospective cohort study was conducted at a pediatric dental practice in Hannover (Germany) [16] following STROBE guidelines for methodological rigor [33]. This study aimed to assess the effect of adding ibuprofen to midazolam sedation on pediatric patient behavior during dental procedures. A total of 1885 children were screened between November 2010 and December 2011. Of these, 311 patients aged 24 to 167 months (2–13 years old) met the inclusion criteria, which included the need for dental treatment for carious lesions and an ASA classification of I or II. Exclusions included patients requiring general anesthesia, those treatable without sedation, siblings, and children not attending regular school. Patients who can be treated without sedation are usually treated without additional medication. General anesthesia is usually required when there is a lack of compliance or when very extensive dental treatment is required that cannot be performed with sedation. Siblings were excluded to avoid potential bias because siblings often share similar home environments, anxiety patterns, or learned behaviors, which could potentially influence the study results. Children who do not attend regular school were also excluded because their educational and social experiences may be different from other children, which could affect their cognitive and emotional development modifying their anxiety levels, cooperation during dental treatment, or understanding of dental procedures, leading to inconsistent or unreliable data.

### 2.2. Ibuprofen and Midazolam

Ibuprofen is a propionic acid derivative with the chemical formula C_13_H_18_O_2_ and the IUPAC name 2-(4-isobutylphenyl)propionic acid. Pharmacokinetically, ibuprofen is rapidly absorbed after oral administration, with peak plasma concentrations reached after approximately 1–2 h. Metabolism occurs primarily in the liver by hydroxylation and carboxylation to inactive metabolites, 98% of which are excreted in the urine within 24 h. The plasma half-life is approximately two hours. Pharmacodynamically, ibuprofen inhibits the enzymes cyclooxygenase 1 (COX-1) and cyclooxygenase 2 (COX-2), resulting in reduced synthesis of prostaglandins and thromboxanes. This results in anti-inflammatory, analgesic, and antipyretic effects (https://pubchem.ncbi.nlm.nih.gov/compound/Ibuprofen; last accessed 14 October 2024).

Midazolam is a benzodiazepine with the chemical formula C_18_H_13_ClFN_3_ and the IUPAC name 8-chloro-6-(2-fluorophenyl)-1-methylimidazo [1,5-a][1,4]benzodiazepine. Pharmacokinetically, midazolam is rapidly absorbed, with peak concentrations reached after 1–2 h when taken orally. It is mainly metabolized in the liver to 1-hydroxymidazolam by the enzyme CYP3A4. Most are excreted in the urine and have a half-life of approximately 1.5–3 h. Pharmacodynamically, midazolam acts as an agonist at the GABA-A receptor, enhancing the inhibitory effects of GABA to produce sedative, anxiolytic, and anticonvulsant effects (https://pubchem.ncbi.nlm.nih.gov/compound/Midazolam; last accessed 14 October 2024).

### 2.3. Randomization and Intervention

Patients were randomly assigned post hoc to one of two groups: a treatment group receiving a combination of midazolam and ibuprofen 30 min prior to treatment and a control group receiving midazolam alone. Midazolam (Midazolam-ratiopharm^®^ 2 mg/mL orale Lösung, ratiopharm GmbH, Ulm, Germany) and ibuprofen (IBU-ratiopharm^®^ Fiebersaft für Kinder 4%, ratiopharm GmbH, Ulm, Germany) were applied as liquids for children orally.

Midazolam was administered at a dose of 0.4 mg/kg body weight, and ibuprofen was administered on the child’s body weight according to the following list: 10–15 kg: 2.5 mL (=100 mg Ibuprofen); 16–19 kg: 3.75 mL (=150 mg ibuprofen); 20–29 kg: 5.0 mL (=200 mg ibuprofen); 30–39 kg: 7.5 mL (=300 mg ibuprofen); and >40 kg: 5–10 mL (=200–400 mg ibuprofen). The primary outcome, child behavior during the dental procedure, was assessed using the Venham Behavior Rating Scale [34]. All procedures were videotaped for independent, blinded evaluation of the behavior scores [30].

### 2.4. Data Collection and Outcome Measures

Demographic data, ASA classification, dental history, and parental anxiety were recorded. The parent’s report of the child’s (dental) anxiety was measured using the Dental Subscale of Children’s Fear Survey Schedule (CFSS-DS) [35,36] and the Inventory of Stressful Situations (ISS) questionnaires during the procedure [29]. This study’s primary outcome was the behavior of the child, which was recorded at multiple stages of the procedure using the Venham scale [34]. The videotaped sessions were reviewed by an independent dental observer who was blinded to the group assignments, ensuring unbiased behavior assessment. The Wong–Baker FACES^®^ Pain Rating Scale was used to evaluate pain and mood and was validated for pediatric populations in dental settings [3].

### 2.5. Statistical Analysis

Data analysis was performed using IBM SPSS Statistics version 20 (IBM, Armonk, NY, USA). The primary comparison of behavior scores between the two groups was conducted using the Mann–Whitney U test, which is appropriate for ordinal data. Secondary analyses explored correlations between behavioral scores and physiological measures, such as pulse rate and oxygen saturation. Statistical significance was set at *p <* 0.05. This study’s retrospective nature and reliance on parental reports were acknowledged as potential sources of bias, though these were mitigated by this study’s randomized design, blinding, and sample size.

## 3. Results

### 3.1. Sample Characteristics

This study included 311 pediatric patients with a mean age of 74.2 months (standard deviation (SD) = 24.7, range: 26–167 months), of which 155 were allocated to the “midazolam plus ibuprofen” group and 156 to the “midazolam only” group. The gender distribution was 169 boys (54.3%) and 142 girls (45.7%), with no significant difference in mean age between boys (mean = 74.1 months, SD = 24.8) and girls (mean = 74.3 months, SD = 24.6; t(309) = −0.285, *p =* 0.78). The distribution was balanced between the treatment groups. Regarding ethnic background, 223 children (71.7%) were born in Germany, 53 (17.1%) were migrants holding a German passport, and 35 (11.2%) had a non-German passport. The reasons for attending the Magic Dental practice were as follows: 38.5% were referred by their family dentist, 7.1% had siblings or other relatives as patients in the practice, and 23.8% were recommended by family or friends. Concerning previous dental experiences, 78 children (25.1%) had been treated under general anesthesia (GA) at the Magic Dental practice, 17 (5.5%) had received GA elsewhere, 51 (16.4%) had prior treatments by Magic Dental practice dentists without medical support, and 42 (13.5%) reported negative experiences with a family dentist. Parental presence during treatment varied, with 73.6% of parents remaining in the waiting room. Among the 26.4% who accompanied their children into the treatment room, 18.6% sat calmly, 6.6% sought bodily contact, and 1.2% exhibited nervous wandering.

### 3.2. Anxiety Assessments: CFSS-DS and ISS Questionnaires

The Inventory of Stressful Situations (ISS) scores, indicating a general fearful disposition, averaged 29.2 (SD = 7.97), with boys scoring 29.1 (SD = 8.6) and girls 29.3 (SD = 7.2). There was no significant difference between genders (t(308) = −0.285, *p =* 0.78). The Children’s Fear Survey Schedule–Dental Subscale (CFSS-DS) scores, representing dental fear, averaged 37.2 (SD = 10.91), with boys scoring 36.5 (SD = 10.6) and girls 38.1 (SD = 11.2). Gender differences were non-significant (t(307) = −1.266, *p =* 0.21). A total of 105 children (34%) were categorized as non-anxious (CFSS-DS < 32), 62 (20%) as borderline (32 ≤ CFSS-DS ≤ 38), and 144 (46%) as highly anxious (CFSS-DS > 38). Significant differences in anxiety levels were observed based on referral reasons and previous dental experiences. Children referred after unsuccessful treatments or with reported negative dental experiences exhibited higher anxiety compared to those preventively referred (t(170) = −2.123, *p =* 0.04; t(147) = −3.049, *p <* 0.01). Additionally, children without prior invasive dental treatments were significantly less anxious than those with such experiences (t(291) = 2.660, *p <* 0.01). Correlation analysis revealed a moderate positive correlation between CFSS-DS and ISS scores (r = 0.551, *p <* 0.01). There was a very weak correlation between parental dental anxiety and children’s dental anxiety (r = 0.124, *p =* 0.03), as well as between the age of the child and CFSS-DS scores (r = 0.187, *p >* 0.01). No correlation was found between age and ISS scores.

### 3.3. Patient Cooperation with and Without Ibuprofen

The main objective was to determine if adding ibuprofen to midazolam improved child cooperation during dental procedures. Table 1 shows the average Venham scores, which capture the behavior and cooperation of the children in the two treatment groups during different phases of the treatment, while higher Venham scores indicate more disruptive behavior. No significant differences between the groups at any time during the procedure (during drinking, before the procedure, during anesthesia, or after the procedure; *p >* 0.05) were observed. These results suggest that the addition of ibuprofen did not significantly improve the children’s cooperative behavior during the procedure.

### 3.4. Post-Treatment Pain

Table 2 shows the frequency of postoperative pain according to the type of dental procedure. Postoperative pain occurred only in 7.2% of the children who did not receive ibuprofen. This pain was mainly associated with invasive procedures such as steel crown placement, pulpotomies, or extractions. Children who experienced postoperative pain tended to have longer treatment times and more procedures. These results highlight the potential benefits of prophylactic ibuprofen in preventing pain after invasive procedures.

### 3.5. Patient Experience and Physiological Measurements

Table 3 shows the Wong–Baker pain scale, pulse rate, and Venham scores at different stages of treatment. During treatment, Wong–Baker scores increased slightly, indicating an increase in perceived pain, before returning to normal after the procedure. A similar trend was seen for the pulse rate, which increased steadily throughout the day and peaked after the treatment. Venham scores also increased significantly during treatment, indicating increased restlessness or anxiety, before decreasing after treatment.

### 3.6. Correlations and Treatment Success

Table 4 shows significant correlations between the Wong–Baker pain scale, pulse rates, and Venham scores at different stages of treatment. A moderate correlation between post-treatment Wong–Baker and Venham scores suggest that higher pain scores were also associated with higher levels of agitated behavior. Similarly, there was a moderate correlation between heart rate and Venham scores, suggesting an association between increased heart rate and agitated behavior during the procedure.

In 79.7% of cases, treatment goals were fully achieved, with 12.7% exceeding expectations. In 4.7% of cases, treatment was shortened, and in 2.9%, treatment could not be completed.

## 4. Discussion

### 4.1. Study Design and Drug Selection

This study was designed to assess the impact of adding ibuprofen to midazolam sedation in pediatric dental procedures, specifically in a setting where the dentist could manage the entire sedation process independently, without the need for an anesthetist. Midazolam was selected due to its well-documented safety profile for use by dentists without specialist supervision, with the dosage strictly adhering to the highest recommended safe limit of 0.5 mg/kg [9]. The exclusion of more potent sedatives, like ketamine, despite their proven efficacy [37], was due to the necessity of avoiding the additional risks and complexities associated with their use, which require advanced monitoring by an experienced anesthetist. This study’s design was carefully developed in consultation with a seasoned anesthetist, ensuring that the combination of midazolam and ibuprofen would provide a reliable and safe sedation protocol within the constraints of general dentistry practice.

### 4.2. Impact of Ibuprofen on Patient Behavior

A central hypothesis of this study was that the addition of ibuprofen to midazolam sedation would enhance patient cooperation by reducing pain and discomfort during dental procedures. However, the findings showed no significant difference in behavioral outcomes between the ibuprofen plus midazolam group and the midazolam only group, as measured by the Venham Behavior Rating Scale [34]. This contrasts with the positive results reported in non-dental procedures, such as tympanocentesis, where ibuprofen effectively reduced patient discomfort [38]. The discrepancy may be attributed to the inherently higher anxiety levels associated with dental procedures, which involve more invasive and sensory-stimulating activities, such as drilling and injections. These stimuli likely exacerbate anticipatory fear, which may have a more substantial impact on patient behavior than pain itself. This suggests that while analgesia is essential, it is insufficient on its own to manage the behavioral challenges in pediatric dental sedation. Instead, a more comprehensive approach that also targets the neuropsychological factors underlying anticipatory fear is required to optimize treatment outcomes. These findings suggest that rather than increasing the dose of analgesics, an integrative approach that more closely integrates psychological methods, such as hypnosis, may be more effective in controlling both pain and anxiety during invasive procedures while minimizing the use of medications [17].

### 4.3. Role of Hypnosis and Neuropsychological Considerations

The inclusion of hypnosis as an adjunct to midazolam sedation in this study aimed to leverage its potential to mitigate anxiety and enhance patient cooperation by addressing the psychological aspects of fear and pain. Hypnosis has been recognized in the literature as a valuable tool in pediatric dentistry that is capable of reducing anxiety and pain perception [21], although the extent of its efficacy varies across studies. Neuropsychologically, hypnosis may work by modulating the patient’s sensory processing and emotional responses, thereby altering their perception of pain and reducing the overall stress of the dental procedure. In this study, the use of hypnosis likely contributed to the high success rates observed by facilitating a calmer, more manageable state in patients who might otherwise have exhibited disruptive behavior. This aligns with existing research suggesting that hypnosis can serve as a powerful, non-pharmacological adjunct in pediatric dental settings, helping to create a more positive treatment experience by addressing both the physiological and psychological dimensions of dental anxiety [5,16]. Future research should focus on quantifying the specific neuropsychological effects of hypnosis in conjunction with sedation to further establish its role in enhancing pediatric dental care.

### 4.4. Treatment Success and Clinical Implications

The overall treatment success rate of over 90% observed in this study is notably high, particularly considering the young age and often complex dental needs of the patient population. This success is likely due to the effective integration of multiple strategies: the careful selection and administration of sedation, the use of behavioral management techniques, and the adjunctive use of hypnosis. Although the addition of ibuprofen did not significantly impact patient behavior during treatment, this study underscores the importance of a multifaceted approach to pediatric dental sedation that includes both pharmacological and psychological interventions. The high success rates suggest that this combined approach can effectively manage the challenges of pediatric dental care, reducing both anxiety and pain and leading to more positive treatment experiences. Clinically, these findings support the broader implementation of hypnosis as a standard adjunctive technique in pediatric dentistry, particularly in cases where anxiety and fear are significant barriers to successful treatment. Further studies should aim to refine these approaches, optimizing the balance between pharmacological sedation and psychological support to enhance patient outcomes in pediatric dental care.

### 4.5. Neurophysiological Considerations

There is a controversial discussion about the importance of imagination in hypnosis [39]. Especially with children, one would expect young patients to benefit from a high degree of imagination. Hypnosis is particularly beneficial for children because it reduces not only the perception of pain but also anxiety, which leads to better cooperation during treatment. Therefore, clinical hypnosis in children can be a promising method of using a child’s internal resources to build trust using their own imagination and thoughts to manage pain and anxiety. Children show a pronounced willingness to engage in visualization. Their creative imagination is suitable for hypnosis. Hypnotic interventions can help children achieve a relaxed state to cope with pain, anxiety, and other conditions. It is an effective method of using a child’s internal resources to build self-confidence using their own imagination and thoughts to learn various skills. It was shown that children under hypnosis experienced less pain and anxiety during dental procedures than children in the control group, showing that hypnosis is stronger compared to the placebo effect [40]. A successful application of hypnosis in an anxious four-year-old boy who had two primary molars treated with a composite filling was described [41]. The nearly 16 min dental treatment went without complications and without interruptions. It was shown that hypnosis can be used in the dental treatment of carious primary tooth lesions in children. In this specific case, hypnosis was used not only as an adjuvant but as an alternative to local anesthesia. It was also found that communication and the relationship between the dentist and the four-year-old child were improved using hypnosis. There is still a need for high-quality studies on the numerous positive effects that hypnosis has on dental treatment for children. Direct suggestions used with young children should avoid abstract thinking since this skill is not yet mature and still developing. Language should be used that reflects the child’s language development and communication skills [42]. Suggestions should be direct and concrete. However, with more mature and older children, hypnosis can also include indirect suggestions. A highly effective method with children is to use hypnotic inductions that incorporate imaginative stories that match the child’s interests and fantasy world [43]. It is important to remember that giving children long-lasting hypnotic instructions is counterproductive. Children have a much shorter attention span than adults. The average attention span of a 3-year-old child is 6–8 min, of a 4-year-old child 8–12 min, of a 5–6-year-old child 12–18 min, and of a 7–8-year-old child 12–18 min [44]. Younger children very often prefer to have their eyes open, may have a fluctuating level of attention, and have difficulty sitting still for long periods of time. Young children rarely close their eyes. Up to about 10 years of age, they tend to keep their eyes open [24]. Regardless of age, hypnotic suggestions and imagery should be individualized and personalized, promoting feelings of well-being, comfort, and positive change. In this study, midazolam serves as an ideal pre-induction agent for initiating a hypnotic trance, which may help prevent the experience of aversive stimuli during treatment.

### 4.6. Strengths and Limitations of This Study

One of the key strengths of this study is its practical, real-world setting, where the entire sedation process was managed by a dentist without the need for an anesthetist. This design closely mirrors everyday pediatric dental practice and enhances the external validity of the findings. Additionally, the integration of both pharmacological (midazolam and ibuprofen) and psychological (hypnosis) interventions provides a comprehensive approach to understanding and managing pediatric dental anxiety and behavior. However, this study also has several limitations. The retrospective design inherently limits the ability to establish causal relationships, and potential biases related to the non-randomized allocation of hypnosis could affect the outcomes. The reliance on parental reports for some data may introduce recall bias, and the relatively small sample size in the subgroup of patients exposed to hypnosis could limit the generalizability of the findings related to this intervention. Furthermore, this study did not explore long-term outcomes, such as the persistence of reduced anxiety or improved cooperation in future dental visits, which would be valuable for understanding the enduring effects of the interventions tested. Finally, the lack of a placebo control for ibuprofen limits the ability to fully assess its specific effects on patient behavior, and it is separate from the natural course of the dental procedure and the psychological interventions provided. These strengths and limitations should be considered when interpreting this study’s results and when considering future research directions in the management of pediatric dental anxiety and behavior.

## 5. Conclusions

This study evaluated the effectiveness of combining ibuprofen with midazolam sedation in pediatric dentistry, focusing on patient behavior and post-treatment pain. The results showed that ibuprofen did not improve patient behavior, suggesting that managing anticipatory fear and anxiety—through behavioral strategies such as clinical hypnosis—may be more important than simply managing pain. However, ibuprofen reduced post-treatment pain, particularly for procedures such as stainless-steel crowns and extractions, suggesting its value in cases where significant pain is expected. This study highlights the critical role of behavioral management in improving the effectiveness of sedation. Techniques such as clinical hypnosis can improve patient cooperation and treatment outcomes, so it is important to start and end treatment with the patient in a positive emotional state to prevent anxiety and ensure future cooperation. Overall, this study highlights the importance of combining pharmacological sedation with behavioral strategies for optimal outcomes in pediatric dentistry. Further research is required to refine and verify these approaches in a prospective setting.

## Figures and Tables

**Table 1:** Venham scores (mean, SD) comparing midazolam only vs. midazolam + ibuprofen.

Treatment Stage	Midazolam Only	Midazolam + Ibuprofen	*p*-Value
Drinking	0.38 (1.02)	0.61 (1.31)	0.13
Before Treatment	0.22 (0.85)	0.25 (0.93)	0.53
Anesthesia	0.96 (1.31)	1.21 (1.55)	0.29
After Treatment	0.38 (0.96)	0.51 (1.22)	0.68

**Table 2:** Post-treatment pain incidence by procedure type.

Treatment Type	No Pain	Pain	Total
Restoration/strip crown (no anesthesia)	42 (10.9%)	-	42
Restoration/strip crown (with anesthesia)	53 (13.8%)	5 (1.3%)	58
Extraction	74 (19.2%)	3 (0.8%)	77
SSC with pulpotomy/root canal	107 (27.8%)	16 (4.2%)	123
Other	109 (28.3%)	8 (2.1%)	117

**Table 3:** Wong–Baker, pulse rate, and Venham scores across treatment stages.

Stage	Wong–Baker (Mean, SD)	Pulse Rate (Mean, SD)	Venham (Mean, SD)
Drinking	1.93 (1.13)	88.31 (13.94)	0.49 (1.18)
Before Treatment	2.10 (1.34)	87.65 (13.91)	0.23 (0.89)
During Treatment	2.22 (1.58)	95.60 (18.51)	1.19 (1.13)
After Treatment	1.74 (1.20)	102.30 (16.19)	0.44 (1.10)

**Table 4:** Significant correlations between Wong–Baker, pulse rate, and Venham scores.

Correlation	Drinking	Before Treatment	After Treatment
Wong–Baker and Venham	0.18	0.11	0.43
Pulse and Venham	0.23	0.16	0.34
Pulse and Wong–Baker	0.05	0.05	0.15

## Data Availability

The data analyzed in this study are subject to the following licenses/restrictions: General European Data Protection Regulation (GDPR) of 25 May 2018. Requests to access these datasets should be directed to the corresponding author.
